# Supplementary material for: Generation of “OP7 chimera” defective interfering influenza A particle preparations free of infectious virus that show antiviral efficacy in mice
Source: Sci Rep. 2023 Nov 28;13:20936. doi: 10.1038/s41598-023-47547-1 (PMC10684881; doi:10.1038/s41598-023-47547-1)
Supplement: Supplementary file 1 — Supplementary Figure 1. [file 41598_2023_47547_MOESM1_ESM.docx]

**Supplementary information of the article:**

**Generation of “OP7 chimera” defective interfering influenza A particle preparations free of infectious virus that show antiviral efficacy in mice**

**Tanya Dogra^1^, Lars Pelz^1^, Julia D. Boehme^2,3^, Jan Kuechler^1^, Olivia Kershaw^4^, Pavel Marichal-Gallardo^1^, Maike Baelkner^2,3^, Marc D. Hein^5^, Achim D. Gruber^4^, Dirk Benndorf^1,5^, Yvonne Genzel^1^, Dunja Bruder^2,3^, Sascha Y. Kupke^1,*^, Udo Reichl^1,5^**

^1^Max Planck Institute for Dynamics of Complex Technical Systems, Bioprocess Engineering, Magdeburg, Germany

^2^Institute of Medical Microbiology, Infection Prevention and Control, Infection Immunology Group, Health Campus Immunology, Infectiology and Inflammation, Otto von Guericke University Magdeburg, Magdeburg, Germany

^3^Immune Regulation Group, Helmholtz Centre for Infection Research, Braunschweig, Germany

^4^Freie Universität Berlin, Department of Veterinary Pathology, Berlin, Germany

^5^Otto von Guericke University Magdeburg, Bioprocess Engineering, Magdeburg, Germany

*** Correspondence:**

Sascha Y. Kupke

[kupke@mpi-magdeburg.mpg.de](mailto:kupke@mpi-magdeburg.mpg.de)


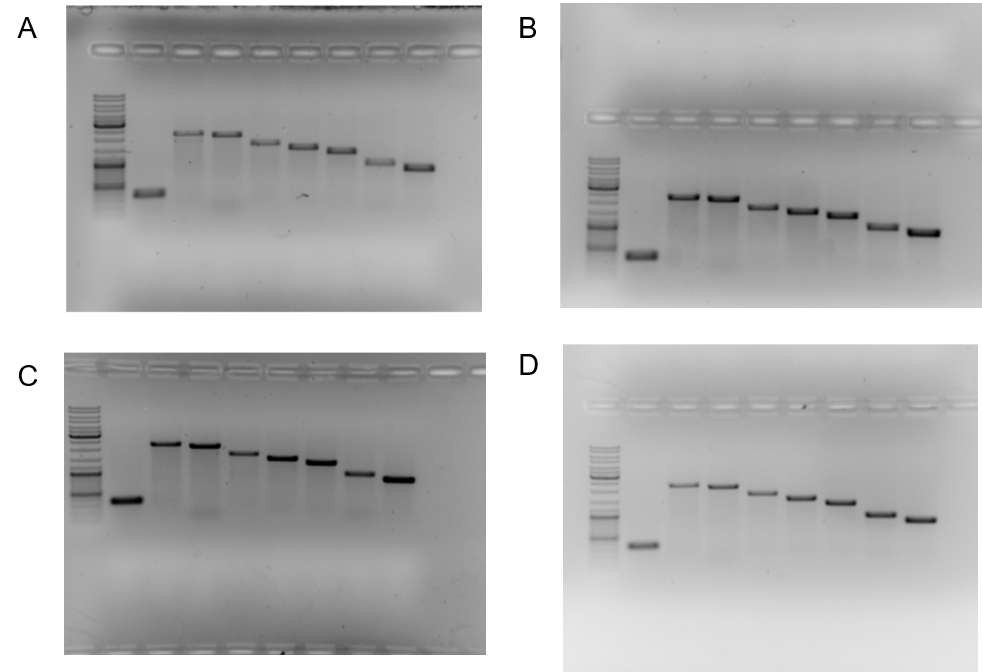


**Supplementary Figure 1: Original images of cropped gels presented in Figure 3.** **(A**) MOI 1E‑2, (**B**) MOI 1E‑3, (**C**) MOI 1E‑4, and (**D**) MOI 1E‑5. Captured using BioDocAnalyze from Biometra.
